# Impact of Procedural-Imaging Configurations on Radiation Dose During Endovascular Flow Diverter Treatment for Intracranial Aneurysms: A Comparison Between Hybrid Operating Room and Neuroangiography Suite

**DOI:** 10.3390/biomedicines14061247

**Published:** 2026-05-30

**Authors:** Kuo-Wei Chen, Yu-Cheng Huang, Yen-Heng Lin, Chung-Wei Lee

**Affiliations:** 1 Division of Neurosurgery, Department of Surgery, National Taiwan University Hospital, Taipei City 100225, Taiwan; duckgoway@gmail.com; 2 Department of Medical Imaging, National Taiwan University Hospital, Taipei City 100225, Taiwanrad.chungweilee@gmail.com (C.-W.L.); 3 Department of Medical Imaging, National Taiwan University Cancer Center, Taipei City 100225, Taiwan

**Keywords:** radiation exposure, aneurysm, flow diverter

## Abstract

**Background and Purpose**: The integration of flow diverter (FD) treatment into hybrid operating rooms (HORs) raises concerns regarding radiation safety, especially when transitioning from biplane systems to single-plane configurations. In this study, we evaluated the impact of distinct procedural-imaging configurations on patient radiation exposure during FD treatment for unruptured cerebral aneurysms. **Methods**: We retrospectively reviewed 93 patients (HOR: 22; biplane neuroangiography suite [NIS]: 71) treated between 2020 and 2024. Key metrics included fluoroscopy time (FT) and dose area product (DAP), subdivided into 2D fluoroscopy and 3D rotational angiography (3D-RA). Linear regression was used to identify independent predictors of radiation dose. **Results**: While the HOR significantly reduced fluoroscopy time (19.3 vs. 26.1 min, *p* = 0.002), it was associated with a higher total DAP compared to the NIS (299.1 vs. 96.3 Gy·cm^2^, *p* < 0.001). This increase was primarily driven by a substantially higher radiation dose delivered per 3D-RA acquisition in the HOR environment rather than an increased frequency of 3D imaging. Multivariate analysis confirmed that the surgical imaging configuration was the dominant factor influencing total radiation exposure rather than aneurysm complexity or patient characteristics. **Conclusions**: Hybrid ORs provide procedural efficiency but involve a significant risk of increased radiation dose due to the reliance on 3D imaging for single-plane navigation. These findings serve as preliminary institutional benchmark data, underscoring the need for adaptive radiation management and configuration-specific protocols to optimize patient safety across diverse surgical imaging suites.

## 1. Introduction

The endovascular treatment of intracranial aneurysms has undergone a paradigm shift with the advent of flow diverters (FDs), which offer a highly refined and effective alternative to traditional intrasaccular techniques [1]. Due to the versatility of FDs, their clinical application, though initially reserved for complex or large aneurysms, has expanded to the routine management of small-to-medium unruptured aneurysms, characterized by high procedural success and favorable safety profiles [2,3]. As FD procedures become increasingly prevalent, there is a growing trend among multidisciplinary teams to perform these interventions within hybrid operating rooms (HORs), which integrate advanced surgical capabilities with high-end imaging technology to cater to operators across both surgical and endovascular disciplines [4].

A critical component of endovascular safety is the establishment of diagnostic reference levels (DRLs), which provide essential benchmarks for monitoring radiation exposure [5]. While DRLs are well documented for conventional coiling procedures, there is an urgent need for updated data that reflect the use of modern devices like FDs in evolving surgical landscapes [6]. Despite the rapid adoption of hybrid operating rooms (HORs), which integrate advanced surgical and imaging tools, systematic research on their application within the neurointerventional field remains notably limited. Consequently, the unique operational workflow of an HOR presents distinct challenges for radiation management, and comprehensive dosimetric data specifically comparing these environments to neuroangiography suites are lacking [6]. Furthermore, the existing literature has predominantly emphasized occupational radiation risks to the surgical team, leaving a paucity of comprehensive data regarding patient-specific radiation doses during complex neurointerventional procedures [7,8,9]. Addressing this gap is essential for establishing standardized procedural practices and ensuring optimized patient safety across diverse surgical settings.

In this retrospective study, in order to address these gaps, we compared radiation exposure during FD treatment for small-to-medium unruptured aneurysms in two environments: a biplane neuroangiography suite (NIS) and a single-plane hybrid operating room (HOR). We analyzed the specific dose contributions of fluoroscopy, two-dimensional (2D) digital subtraction angiography, and three-dimensional (3D) rotational angiography to identify key procedural drivers of radiation exposure. Our findings support the establishment of evidence-based DRLs and optimized imaging protocols for patient safety in contemporary neurointervention.

## 2. Materials and Methods

### 2.1. Study Participants

**Figure 1:**
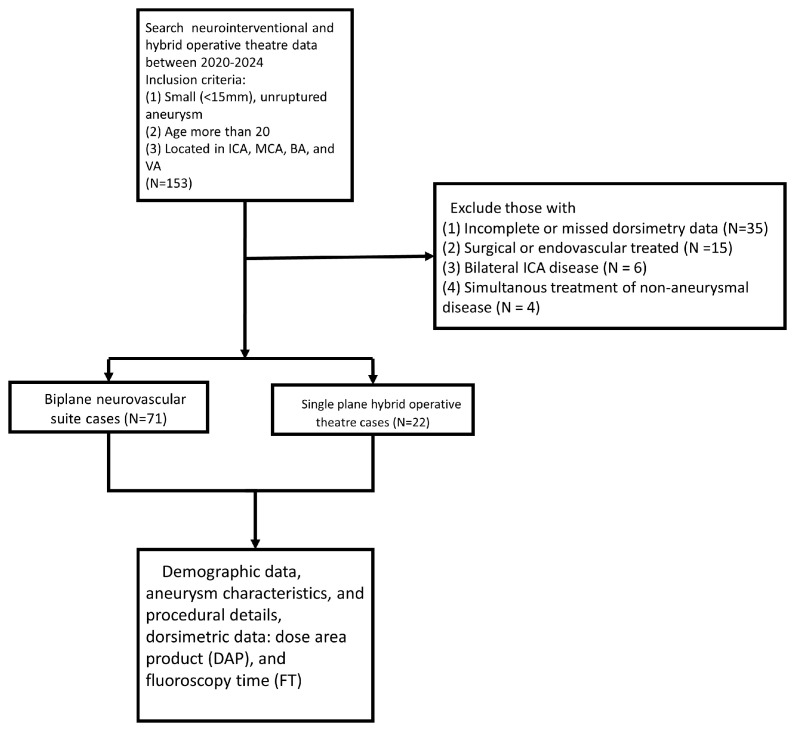
Flowchart of study cohort selection process.

This single-center retrospective study was approved by the Institutional Review Board of National Taiwan University Hospital (Approval number: 202407146RINA), with a waiver of individual informed consent. The study followed the STROBE guidelines and included patients with small-to-medium unruptured aneurysms (saccular or fusiform, maximum diameter < 15 mm) treated with FDs between January 2020 and June 2024. The inclusion criteria were as follows: (1) age ≥ 20 years and (2) aneurysms located in the anterior circulation (internal carotid or middle cerebral artery) or posterior circulation (basilar or vertebral artery). Exclusion criteria included (1) incomplete or missing dosimetric data, (2) recurrent aneurysms after prior therapy, (3) bilateral internal carotid artery disease, or (4) simultaneous endovascular treatment for unrelated conditions. The study flowchart is presented in Figure 1.

### 2.2. Endovascular Treatment

All procedures were performed by a single neurointerventional team comprising two neuroradiologists and one neurosurgeon to ensure technical consistency. Vascular access was obtained via the common femoral or radial artery. The standard workflow was initiated with two-dimensional (2D) digital subtraction angiography (DSA) in frontal and lateral projections. Three-dimensional (3D) rotational angiography was routinely performed for precise vessel and aneurysm measurements to facilitate device sizing, unless recent 3D data were already available. Intraprocedural monitoring utilized fluoroscopy with roadmapping guidance, supplemented by intermittent 2D-DSA to verify the location and expansion of the flow diverter (FD). Intraoperative 3D rotational angiography was selectively employed if uncertainties arose regarding optimal device opening or positioning. Following complete deployment, standard 2D-DSA projections were repeated to match preoperative images. Finally, high-resolution post-deployment 3D rotational angiography was mandatory to confirm precise vascular wall apposition and ensure procedural success.

### 2.3. Imaging System and Radiation Metrics

Procedures were performed using either a biplane angiography system (Artis Q; Siemens Healthineers, Erlangen, Germany) in the neuroangiography suite (NIS) or a single-plane angiography system (Artis Pheno; Siemens Healthineers, Erlangen, Germany) in the hybrid operating room (HOR). Both systems were equipped with 20-inch detectors offering a maximum field of view of 48 cm. The specific imaging protocols for each environment were configured as follows:

#### 2.3.1. Neuroangiography Suite (NIS: Artis Q)

- Fluoroscopy: Pulsed mode at 7.5 pulses/s (36 nGy/pulse, 81 kV, pulse width 12.5 ms).
- 2D digital subtraction angiography (DSA): Acquisition rates of 4 frames/s (0–4 s), 2 frames/s (4–11 s), and 1 frame/s thereafter (73 kV, pulse width 64 ms, dose 1.820 μGy/frame).
- 3D rotational angiography (subtraction): Acquisition rate 1.5°/frame (70 kV, dose 0.360 μGy/frame, pulse width 12.5 ms).
- Post-deployment 3D rotational angiography (high-resolution): Acquisition rate 0.8°/frame (70 kV, dose 1.2 μGy/frame, pulse width 12.5 ms).

#### 2.3.2. Hybrid Operating Room (HOR: Artis Pheno)

- Fluoroscopy: Pulsed mode at 7.5–15 pulses/s (40 nGy/pulse, 73 kV, pulse width 12.5 ms).
- 2D digital subtraction angiography (DSA): Acquisition rates of 4 frames/s (0–4 s), 2 frames/s (4–11 s), and 1 frame/s thereafter (73 kV, pulse width 125 ms, dose 2.400 μGy/frame).
- 3D rotational angiography (subtraction): Acquisition rate 0.5°/frame (70 kV, dose 1.2 μGy/frame, pulse width 8 ms).
- Post-deployment 3D rotational angiography (high-resolution): Acquisition rate 0.4°/frame (109 kV, dose 1.82 μGy/frame, pulse width 8 ms).

The following dosimetric parameters were documented: DSA acquisition count, fluoroscopy time, and dose area product (DAP). Total DAP was calculated by summing the contributions from fluoroscopy and all DSA acquisitions. For biplane systems, DAP from both X-ray tubes was aggregated, and simultaneous biplane acquisitions were counted as two distinct events. The mean dose index was calculated by dividing the total DAP by the total acquisition count for both 2D and 3D angiography.

### 2.4. Statistical Analysis

Descriptive statistics were used to summarize clinical data and aneurysm characteristics, with continuous variables expressed as means ± standard deviations and categorical variables as counts and percentages. Comparative analyses between the HOR and neuroangiography suite were performed using Student’s *t*-test, the Mann–Whitney U test, or the chi-square test, as appropriate for the data distribution. For radiation metrics, such as DAP and fluoroscopy time, both mean (SD) and median (interquartile range) values were reported to determine the 75th percentile benchmarks. DAP data from 2D-DSA and 3D rotational angiography were analyzed independently.

Univariate and multivariate linear regression analyses were performed to identify independent predictors of radiation dose metrics and fluoroscopy time. Logarithmic transformation was applied to all dependent variables to correct for skewed distributions and ensure the normality of residuals. To rigorously control for confounding, the final multivariate models simultaneously incorporated important clinical, morphological, and procedural variables (including age, aneurysm size, type, location, device type [pipeline embolization device vs. non-pipeline embolization device], and imaging configurations). Overall model fit and explanatory power were evaluated using the coefficient of determination (model R^2^), and 95% confidence intervals (CIs) were reported for all estimates. All statistical analyses were conducted using SAS 9.4 (SAS Institute, Cary, NC, USA), with a two-tailed *p*-value < 0.05 considered statistically significant.

## 3. Results

### 3.1. Baseline Clinical and Aneurysmal Characteristics

The study cohort included 93 patients with a mean age of 58.5 years, including 25 (27%) men. Among these patients, 71 underwent procedures (76%) conducted in a biplane neuroangiography suite (NIS), while 22 (24%) were treated in a single-plane hybrid operating room (HOR). Aneurysms were primarily located in the internal carotid artery (n = 71, 77%), followed by the vertebral artery (n = 18, 19%) and middle cerebral artery (n = 4, 4%). The mean maximum aneurysm diameter was 5.6 mm, with a morphological distribution of 75 saccular (81%) and 18 fusiform (19%) aneurysms. The implanted devices had a mean diameter of 4.3 mm and a mean length of 17.4 mm.

Comparative analysis revealed that patient and aneurysm characteristics were well balanced between the NIS and HOR environments (Table 1). The only statistically significant difference was the type of flow diverter utilized (*p* < 0.001); specifically, the Surpass Evolve device was used in 68% of HOR cases compared to 33% in the NIS. Regarding procedural adjuncts, balloon angioplasty and rescue stenting for device shortening were each performed in one case per environment. While minor complications in the HOR group included one internal carotid artery dissection and one radial wire dislodgement, no periprocedural neurological complications were observed in either group.

### 3.2. Comparative Analysis of Radiation Metrics and Fluoroscopy Time

**Figure 2:**
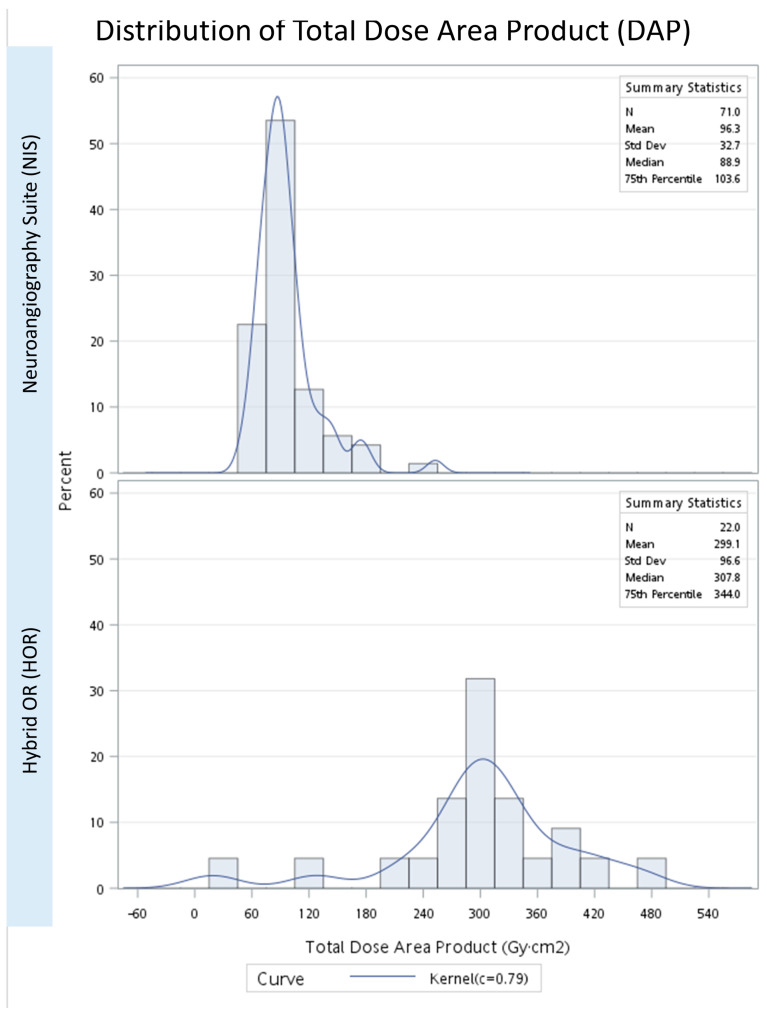
Histogram distribution of total dose area product (DAP) in the neuroangiography suite and hybrid operating room.

Radiation dose metrics and fluoroscopy times were evaluated for all participants, with significant differences observed between the two procedural environments (Table 2 and Figure 2). The mean total DAP was significantly higher in the HOR than in the NIS (299.1 vs. 96.3 Gy·cm^2^, *p* < 0.001), a trend consistently reflected in the median values (307.8 vs. 88.9 Gy·cm^2^, *p* < 0.001). Conversely, the HOR was associated with significantly shorter fluoroscopy times compared to the NIS (mean: 19.3 vs. 26.1 min, *p* = 0.002; median: 18.2 vs. 23.6 min, *p* = 0.004).

The analysis of two-dimensional (2D) DSA revealed that, while the acquisition count was significantly lower in the HOR (7.8 vs. 13.1, *p* < 0.001), the associated DAP and mean dose index were both significantly higher (*p* < 0.001). Regarding three-dimensional (3D) rotational angiography, there was no significant difference in the acquisition count between the HOR and NIS (3.1 vs. 2.8, *p* = 0.23). However, both the mean DAP (240.7 vs. 45.5 Gy·cm^2^) and the mean dose index (75.6 vs. 17.4 Gy·cm^2^) for 3D rotational angiography were markedly elevated in the HOR environment (*p* < 0.001). Furthermore, the mean DAP during fluoroscopy was also higher in the HOR (34.1 vs. 18.6 Gy·cm^2^, *p* = 0.003).

### 3.3. Factors Associated with Radiation Exposure and Fluoroscopy Time

To identify independent predictors of radiation dose and procedural efficiency, univariate and multivariate linear regression analyses were performed (Table 3). Within the final multivariate framework, the imaging configuration emerged as the single dominant independent predictor: performing procedures in the HOR was independently associated with a higher total DAP and 2D DAP, but shorter fluoroscopy times (all *p* < 0.05). In contrast, patient characteristics, morphological factors (including age, aneurysm size, type, and location), and device type (pipeline embolization device vs. non-pipeline embolization device) did not significantly influence cumulative radiation exposure. The final multivariate models demonstrated strong explanatory power for the total DAP (model R^2^ = 0.5795, *p* < 0.0001).

Subgroup analyses of aneurysm characteristics (available in Supplementary Materials, Table S1) indicated that saccular and anterior circulation aneurysms were associated with a lower 2D DAP (*p* = 0.01 and *p* < 0.001, respectively). However, these factors did not translate into a significant reduction in the cumulative radiation dose (total DAP), further reinforcing the dominant impact of the surgical environment and its associated imaging protocols on overall patient exposure.

Model Fit Statistics (Multivariate Framework).

- Total DAP model: Adjusted for all listed predictors; overall model R^2^ = 0.5795, *p* < 0.0001.
- Fluoroscopy time model: Adjusted for all listed predictors; overall model R^2^ = 0.1283, *p* = 0.0469.
- 2D-DSA DAP model: Adjusted for all listed predictors; overall model R^2^ = 0.2540, *p* = 0.0003.

## 4. Discussion

This study was conducted at a single center with the same surgical team operating across two distinct clinical environments, allowing for a controlled comparison by minimizing inter-operator technical variance. By isolating the environmental variable, our analysis yielded several key findings regarding radiation safety during flow diverter (FD) treatment. First, differences in machine settings between the environments significantly influenced radiation exposure; specifically, biplane angiography settings utilizing optimized low-dose protocols were associated with substantially lower radiation exposure when compared with the default standard-dose settings often pre-configured in hybrid suites. Second, radiation doses from rotational angiography contributed significantly to the overall cumulative exposure in both environments, though their impact was disproportionately higher in the single-plane setting. Third, for small-to-medium unruptured aneurysms, radiation doses associated with FD procedures were slightly lower for anterior circulation aneurysms than for posterior circulation aneurysms, likely due to anatomical rather than procedural factors. Finally, despite the observed reduction in fluoroscopy time in the single-plane HOR, this procedural efficiency did not offset the significantly higher total radiation dose recorded in this environment.

In the study cohort, the average DAP was 144.3 Gy·cm^2^ (median: 95.8 Gy·cm^2^; 75th percentile: 169.6 Gy·cm^2^), with a mean fluoroscopy time of 24.5 min (75th percentile: 28.5 min). Although established guidelines have previously reported a diagnostic reference level (DRL) of 250 Gy·cm^2^ for generic aneurysm treatments [10], subsequent research has documented a consistent decreasing trend, with lower radiation doses reported specifically for unruptured aneurysms treated with flow diverters [11,12,13,14]. While our overall findings align with this global trend toward radiation reduction, a significant discrepancy remains between different procedural settings: the 75th percentile DAP in the HOR (343.9 Gy·cm^2^) was substantially higher than in the neuroangiography suite (103.6 Gy·cm^2^) and exceeded traditional DRL thresholds. Although this disparity underscores the critical role of imaging configurations, given our single-center retrospective design and the modest HOR cohort size, these findings should not be interpreted as definitive reference levels. Instead, they provide valuable preliminary institutional benchmark data that can serve as a localized reference for dose monitoring and guide future multicenter registry studies aimed at defining standardized benchmarks. The higher exposure observed in the HOR is largely attributed to the standard higher-dose DSA settings frequently utilized to ensure optimal visualization of the FD mesh within complex surgical fields, highlighting the necessity of balancing image quality with site-specific dose optimization.

Rotational angiography was identified as a major contributor to radiation exposure in this study, accounting for 80.1% of the total radiation exposure in the HOR compared to 47.2% in the neuroangiography suite. This difference highlights the disproportionate impact of rotational angiography on overall exposure, particularly within HOR environments. Although fluoroscopy time was notably shorter in the HOR, this reduction did not compensate for the higher total radiation dose, which was predominantly driven by the increased average doses associated with rotational angiography. Notably, the number of 3D-RA acquisitions did not significantly differ between the two groups (3.0 vs. 2.8, *p* = 0.38), implying that the cumulative dose discrepancy is primarily driven by a system-inherent factor: the remarkably higher radiation dose per single 3D-RA scan in the HOR system. Several technical and geometric factors may explain this elevated dose per 3D acquisition in the HOR. First, the HOR utilized a single-plane system optimized for combined open-surgical and endovascular workflows, which often involve different automatic brightness control (ABC) baseline algorithms and pulse configurations compared to a dedicated biplane NIS. Second, to clear the surgical table and anesthesia equipment, the gantry geometry and source-to-image distance (SID) in an HOR might be automatically maximized during a rotational cone-beam CT sweep, forcing the X-ray tube to output higher mAs/kVp to maintain image quality through automatic exposure control. These configuration differences result in a steeper dose delivery per rotation, even when the absolute number of scans remains unchanged. Future research should continue to explore imaging strategies that maintain these rigorous safety standards while reducing the overall reliance on high-dose techniques, thereby enhancing both patient and operator safety in complex neurointerventional procedures.

For small-to-medium aneurysms, while the impact of factors such as location and morphology on radiation dose was relatively minimal, slightly higher radiation doses were identified for fusiform aneurysms and posterior circulation aneurysms. These subtle dosimetric trends may be partially explained by regional anatomical characteristics rather than increased procedural difficulty, given that fluoroscopy times for these cases remained notably shorter. It is speculated that the higher radiopacity of skull base structures—such as the clivus and petrous bone—causes greater X-ray attenuation during posterior circulation imaging. This could potentially prompt the system’s automatic brightness control (ABC) algorithm to baseline at a higher tube voltage or current to preserve image quality. Although the lack of intraoperative tube parameter logging limits our ability to directly test this mechanism, such system-driven adjustments in anatomically dense regions represent a plausible contributing factor. Advanced filtration or customized low-dose baselines may help mitigate these potential anatomical variances, though further investigation in larger cohorts is needed to confirm these technical mechanisms [15,16]. Future research with larger, multicenter cohorts will be essential to validate these findings and explore effective strategies for mitigating radiation exposure in anatomically complex scenarios [17].

In our study, we demonstrated that the biplane NIS achieved notably lower radiation doses, which can be directly elucidated by the distinct operational governance and protocol optimization between the two environments. At our institution, the biplane NIS functions as a dedicated neuroradiology-specific suite. During its initial installation, our radiological team actively optimized the system parameters to the lowest radiation thresholds acceptable to the primary operators. The literature confirms that such tailored refinements—including reducing the pulse rates and enhancing spectral filtration—can lower cumulative exposure by 20–43% [12,18,19]. In contrast, the robotic HOR operates as a shared, multidisciplinary facility utilized by multiple surgical disciplines. To accommodate the heterogeneous visualization habits of various surgical teams without disrupting cross-departmental workflows, the HOR system predominantly relies on standard factory-default settings. Consequently, while modifying the HOR imaging protocols toward a low-dose direction is technically straightforward via system recalibration, its implementation in real-world practice faces pragmatic institutional constraints. Collaborative, multi-specialty radiation governance is required to modify shared HOR baselines safely, ensuring that dose-reduction strategies do not compromise the stringent visualization demands across different surgical portfolios [8,20]. Furthermore, the success of intricate neurointerventional procedures under tailored imaging configurations heavily relies on strict protocol compliance and streamlined team coordination, as highlighted by recent retrospective cohort studies addressing workflow optimization in neurovascular centers [21]. Ultimately, adhering to the “As Low As Reasonably Achievable” (ALARA) principle remains critical, as efforts to minimize both radiation and contrast agent usage are essential to mitigate systemic risks, including contrast-induced nephropathy and radiation-induced malignancies [22].

In this study, we systematically evaluated radiation exposure associated with FD procedures in different clinical environments, offering critical preliminary institutional benchmark data regarding the transition to robotic hybrid suites. However, several limitations should be considered when interpreting these findings. First, the retrospective nature of the study may have introduced selection bias, as only cases with complete radiation dose data were included, potentially affecting the generalizability of the results. Additionally, the relatively small sample size, particularly in the HOR cohort of 22 cases, may have limited the statistical power to detect subtle differences between settings. Second, the study was conducted at a single center with a consistent operator team. Although temporal bias or chronological variations in operator proficiency could have potentially influenced radiation and fluoroscopy metrics, our institutional team had already accumulated a high volume of experience prior to the study’s inclusion period. Therefore, while individual learning curves were not explicitly modeled, technical proficiency remained stable throughout the study period, though caution should still be exercised when generalizing these single-center findings to institutions with differing operator experience profiles. Third, the focus on small-to-medium, uncomplicated aneurysms allowed for a controlled analysis but did not fully capture the complexities of routine clinical practice, where larger or more morphologically complex aneurysms may necessitate higher radiation doses. Additionally, specific anatomical variations, such as extreme vessel tortuosity or challenging proximal catheter navigation, were not quantitatively graded or included in the regression models. However, because all procedures within the study period were performed exclusively using the lower-profile Pipeline Flex system or other modern devices, technical challenges related to device navigation were inherently minimized compared to early-generation devices. Fourth, due to the retrospective nature of this study, objective or subjective image quality assessments were not performed. Consequently, we could not directly evaluate whether the higher radiation dose in the HOR translated into superior procedural visualization, though the technical success rate was identical between the two environments. Fifth, this study focused exclusively on patient radiation metrics (DAP and fluoroscopy time) and did not include measurements of operator or staff radiation exposure. In a hybrid operating room environment, occupational exposure from scatter radiation can vary significantly compared to conventional angiography suites due to differing room configurations, shielding setups, and single-plane gantry positioning. The lack of objective personal dosimeter data for the medical team represents a limitation that should be addressed in prospective future investigations. Finally, it is important to clarify that this study compared two holistic procedural-imaging configurations rather than isolating the effect of the physical surgical environment alone. Consequently, the observed dosimetric discrepancies represent the combined impact of system geometry, hardware-specific baseline outputs, and environmental workflows. Future prospective studies utilizing identical imaging systems across different surgical spaces are required to completely isolate the environmental factor. Despite these limitations, our findings provide a critical baseline for understanding how procedural environments influence radiation profiles during the transition to hybrid operating rooms. Future research with larger, multicenter cohorts is necessary to validate these findings and guide the establishment of environment-specific benchmarks that ensure both procedural efficacy and radiation safety across diverse interventional settings.

## 5. Conclusions

Through this study, we have established a radiation benchmark for the treatment of small-to-medium unruptured aneurysms using flow diverters. We found that, despite lower fluoroscopy times, the single-plane HOR incurred higher total radiation doses, driven primarily by a higher system-specific radiation dose output per 3D rotational angiography acquisition. Furthermore, the increased exposure in posterior circulation and fusiform cases appears to be linked to anatomical factors rather than procedural complexity. These findings highlight the need for environment-specific DRLs and localized guidelines to optimize radiation safety across different procedural settings.

## Figures and Tables

**Table 1:** Characteristics of patients and aneurysms.

		Total (n = 93)	Hybrid OR (n = 22)	Neuroangiographic Suite (n = 71)	*p*-Value
Patient characteristics	Age, mean (SD), y	58.5 (11.9)	56.3 (9.8)	59.2 (12.5)	0.33
	Male sex, n (%)	25 (27)	5 (23)	20 (28)	0.61
Aneurysm location	ICA, n (%)	71 (77)	16 (73)	55 (77)	0.65
	MCA, n (%)	4 (4)	0 (0)	4 (6)	0.57
	VA, n (%)	18 (19)	6 (27)	12 (17)	0.28
Aneurysm size	Diameter, mean (SD), mm	5.6 (2.2)	5.5 (2.1)	5.6 (2.2)	0.76
	Small (<5mm), n (%)	34 (37)	10 (45)	24 (34)	0.67
	Medium (5–10mm), n (%)	53 (57)	11 (50)	42 (59)	
	Large (≥10), n (%)	6 (6)	1 (5)	5 (7)	
Aneurysm type	Saccular, n (%)	75 (81)	16 (73)	59 (83)	0.28
	Fusiform, n (%)	18 (19)	6 (27)	12 (17)	
Device	Pipeline, n (%)	52 (56)	7 (32)	45 (63)	<0.001
	Surpass Evolve, n (%)	38 (41)	15 (68)	23 (33)	
	p64, n (%)	3 (3)	0 (0)	3 (4)	
Device size	Diameter, mean (SD), mm	4.3 (0.6)	4.3 (0.3)	4.3 (0.6)	0.49
	Length, mean (SD), mm	17.4 (4.1)	17.6 (5.2)	17.4 (3.7)	0.84

Abbreviations: OR, operating room; SD, standard deviation; ICA, internal carotid artery; MCA, middle cerebral artery; VA, vertebral artery.

**Table 2:** Radiation dose and fluoroscopy time between two environments.

Total	Total (n = 93)	Hybrid OR (n = 22)	Neuroangiographic Suite (n = 71)	*p*-Value
DAP, mean (SD), [Gy cm^2^]	144.3 (102.2)	299.1 (96.6)	96.3 (32.7)	<0.001
DAP, median (IQR), [Gy cm^2^]	95.8 (81.1–169.6)	307.8 (276.9–343.9)	88.9 (76.9–103.6)	<0.001
2D DSA				
Acquisition count, mean (SD)	11.8 (4.6)	7.8 (2.7)	13.1 (4.4)	<0.001
DAP, mean (SD), [Gy cm^2^]	35.9 (19.2)	48.5 (24.4)	31.9 (15.5)	<0.001
Mean dose index, mean (SD), [Gy cm^2^]	3.4 (2.1)	6.2 (2.4)	2.5 (0.9)	<0.001
3D rotational angiography				
Acquisition count, n (SD)	2.9 (1.0)	3.1 (1.1)	2.8 (0.9)	0.23
DAP, mean (SD), [Gy cm^2^]	52.6 (31.5)	240.7 (95.3)	45.5 (22.7)	<0.001
Mean dose index, mean (SD), [Gy cm^2^]	31.1 (26.1)	75.6 (7.3)	17.4 (8.1)	<0.001
Fluoroscopy				
FL time, mean (SD), [minutes]	24.5 (12.1)	19.3 (6.8)	26.1 (12.9)	0.002
FL time, median (IQR), [minutes]	20.9 (16.1–28.5)	18.2 (15–20.9)	23.6 (17–30.8)	0.004
Mean DAP, mean (SD), [Gy cm^2^]	22.2 (16.6)	34.1 (25.8)	18.6 (10.2)	0.003

Abbreviations: OR, operating room; SD, standard deviation; DAP, dose area product; IQR, interquartile range; FL, fluoroscopy.

**Table 3:** Linear regression analysis results for factors influencing dose area product and fluoroscopy time.

		Univariate Analysis				Multivariate Analysis			
	Predictors	β	SE	95% CI	*p*-Value	β	SE	95% CI	*p*-Value
DAP [Gy cm^2^]—total	Age (years)	−0.0035	0.0053	(−0.0139, 0.0069)	0.5076				
	Aneurysm size (mm)	0.0148	0.0293	(−0.0427, 0.0723)	0.6141	0.0004	0.004	(−0.0075, 0.0083)	0.9077
	Aneurysm type (saccular vs. fusiform)	−0.2797	0.1569	(−0.5872, 0.0279)	0.078	0.0111	0.0229	(−0.0343, 0.0566)	0.6279
	Location (anterior vs. posterior)	−0.2898	0.1567	(−0.5970, 0.0173)	0.0676	−0.015	0.2177	(−0.4478, 0.4178)	0.945
	Environment (hybrid OR vs. NIS)	1.0638	0.0979	(0.8719, 1.2556)	<0.0001	−0.1127	0.2135	(−0.5372, 0.3117)	0.599
	Device type (PED vs. non-PED)	−0.1786	0.1256	(−0.4248, 0.0676)	0.1586	1.0722	0.1056	(0.8622, 1.2822)	<0.0001
FL time [minutes]	Age (years)	0.0083	0.0036	(0.0012, 0.0154)	0.0241	0.0068	0.0036	(−0.0004, 0.0140)	0.0659
	Aneurysm size (mm)	0.0121	0.0205	(−0.0281, 0.0524)	0.5558	0.0114	0.0229	(−0.0340, 0.0568)	0.62
	Aneurysm type (saccular vs. fusiform)	0.0818	0.1115	(−0.1367, 0.3003)	0.4649	0.221	0.2201	(−0.2164, 0.6585)	0.3175
	Location (anterior vs. posterior)	0.0122	0.1118	(−0.2070, 0.2313)	0.9135	−0.1838	0.2155	(−0.6122, 0.2446)	0.3959
	Environment (hybrid OR vs. NIS)	−0.2669	0.1001	(−0.4631, −0.0707)	0.0091	−0.2454	0.1065	(−0.4571, −0.0337)	0.0237
	Device type (PED vs. non-PED)	0.0286	0.0889	(−0.1457, 0.2029)	0.7482	−0.0114	0.0917	(−0.1936, 0.1709)	0.9011
DAP [Gy cm^2^]—2D	Age (years)	−0.0034	0.0037	(−0.0105, 0.0038)	0.3614	−0.0015	0.0033	(−0.0080, 0.0051)	0.6473
	Aneurysm size (mm)	0.0129	0.0203	(−0.0270, 0.0527)	0.5286	−0.0025	0.0208	(−0.0439, 0.0388)	0.9042
	Aneurysm type (saccular vs. fusiform)	−0.2827	0.1066	(−0.4917, −0.0737)	0.0095	−0.0217	0.2005	(−0.4221, 0.3788)	0.914
	Location (anterior vs. posterior)	−0.293	0.1063	(−0.5013, −0.0846)	0.0071	−0.2067	0.1963	(−0.5990, 0.1855)	0.2975
	Environment (hybrid OR vs. NIS)	0.3846	0.0947	(0.1991, 0.5701)	0.0001	0.4123	0.0975	(0.2185, 0.6062)	<0.0001
	Device type (PED vs. non-PED)	0.1039	0.0874	(−0.0674, 0.2752)	0.2375	0.1781	0.0839	(0.0114, 0.3448)	0.0366

Abbreviations: β, regression coefficient; SE, standard error; CI, confidence interval; DAP, dose area product; NIS, neuroangiography suite; hybrid OR, hybrid operating room; PED, pipeline embolization device. Note: Logarithmic transformation was performed on all dependent variables prior to regression analysis to ensure normality of residuals.

## Data Availability

The datasets used and/or analyzed during the current study are available from the corresponding author on reasonable request.

## Supplementary Materials

The following supporting information can be downloaded at https://www.mdpi.com/article/10.3390/biomedicines14061247/s1, Table S1: Dose area product and fluoroscopy time within various categories of aneurysmal characteristics.
