# Supplementary material for: Impact of Procedural-Imaging Configurations on Radiation Dose During Endovascular Flow Diverter Treatment for Intracranial Aneurysms: A Comparison Between Hybrid Operating Room and Neuroangiography Suite
Source: Biomedicines. 2026 May 30;14(6):1247. doi: 10.3390/biomedicines14061247 (PMC13297619; doi:10.3390/biomedicines14061247)
Supplement: Supplementary file 1 [file biomedicines-14-01247-s001.zip › biomedicines-4322007-supplementary.pdf]

Supplementary Table S1. Dose area product and fluoroscopy time within various categories of aneurysmal characteristics, including location and size

	ICA (n=71)	VB (n=18)	MCA (n=4)	P-value
2D-DSA, mean (SD), count	12.0 (4.9)	11.6 (3.8)	10.3(2.3)	0.67
3D rotational angiography, mean (SD), count	3.0 (1.0)	2.6 (1.0)	2.8 (0.5)	0.2
DAP, mean (SD), [Gy cm2]	137.9 (96.5)	183.6 (124)	79.5 (11.6)	0.1
DAP, median (IQR), [Gy cm2]	95.8 (80.0-148.4)	123.7 (87.3-289.1)	83.2 (72.2-86.8)	0.09
FL time, mean (SD), minutes	23.9 (11.2)	24.4 (10.8)	36.8 (25.6)	0.12
FL time, median (IQR), minutes	10.7 (16.1-28.1)	24.3 (15.6-31.3)	40.1 (17.0-56.6)	0.54

	Small (<5mm) (n=34)	Median (5-10mm) (n=54)	Large (>=10) (n=6)	P-value
2D-DSA, mean (SD), count	12.2 (5.0)	11.5 (4.4)	13.0 (4.1)	0.66
3D rotational angiography, mean (SD), count	3.0 (1.1)	2.8 (0.9)	3.0 (0)	0.77
DAP, mean (SD), [Gy cm2]	152.4 (110.6)	139.2 (98.2)	141.8 (102.2)	0.84
DAP, median (IQR), [Gy cm2]	103.2 (79.1-212.2)	91.2 (81.3-136.7)	103.4 (81.1-145.3)	0.36
FL time, mean (SD), minutes	24.3 (11.3)	24.1 (11.7)	29.9 (19.4)	0.53
FL time, median (IQR), minutes	20.9 (16.0-27.5)	20.7 (16.3-28.5)	28.3 (15.0-31.6)	0.68

	Saccular (n=75)	Fusiform (n=18)	P-value
2D-DSA, mean (SD), count	12.1(4.8)	11.0(3.9)	0.39
3D rotational angiography, mean (SD), count	3.0(1.0)	2.4(1.0)	0.01
DAP, mean (SD), [Gy cm2]	134.9(94.7)	183.2(124.6)	0.07
DAP, median (IQR), [Gy cm2]	94.3(80.8-140.0)	131.3(81.1-289.1)	0.27
FL time, mean (SD), [Gy cm2]	24.9(12.4)	23.0(10.5)	0.56
FL time, median (IQR), [Gy cm2]	20.9(16.3-28.5)	22.6(15.0-28.9)	0.57

DAP, dose area product; ICA, internal carotid artery; VB vertebrobasilar artery; MCA, middle cerebral artery; DSA, digital subtraction angiography; SD, standard deviation; IQR, interquartile range. FL, fluoroscopy.
